# Micronutrient Deficiency in Inherited Metabolic Disorders Requiring Diet Regimen: A Brief Critical Review

**DOI:** 10.3390/ijms242317024

**Published:** 2023-11-30

**Authors:** Albina Tummolo, Rosa Carella, Donatella De Giovanni, Giulia Paterno, Simonetta Simonetti, Maria Tolomeo, Piero Leone, Maria Barile

**Affiliations:** 1 Department of Metabolic Diseases, Clinical Genetics and Diabetology, Giovanni XXIII Children Hospital, Azienda Ospedaliero-Universitaria Consorziale, 70126 Bari, Italy; rossycarella@gmail.com (R.C.); degiovanni.dony@gmail.com (D.D.G.); giupatvi@gmail.com (G.P.); 2 Regional Centre for Neonatal Screening, Department of Clinical Pathology and Neonatal Screening, Children’s Hospital “Giovanni XXIII”, Azienda Ospedaliero-Universitaria Consorziale, 70126 Bari, Italy; simonetta.simonetti@policlinico.ba.it; 3 Department of Biosciences, Biotechnology and Environment, University of Bari “A. Moro”, via Orabona 4, 70125 Bari, Italy; maria.tolomeo89@gmail.com (M.T.); piero.leone@uniba.it (P.L.); 4 Department of DiBEST (Biologia, Ecologia e Scienze della Terra), University of Calabria, via P. Bucci 4C, 87036 Arcavacata di Rende, Italy

**Keywords:** micronutrients, oligoelements, vitamins, inherited metabolic disorders, diet therapy

## Abstract

Many inherited metabolic disorders (IMDs), including disorders of amino acid, fatty acid, and carbohydrate metabolism, are treated with a dietary reduction or exclusion of certain macronutrients, putting one at risk of a reduced intake of micronutrients. In this review, we aim to provide available evidence on the most common micronutrient deficits related to specific dietary approaches and on the management of their deficiency, in the meanwhile discussing the main critical points of each nutritional supplementation. The emerging concepts are that a great heterogeneity in clinical practice exists, as well as no univocal evidence on the most common micronutrient abnormalities. In phenylketonuria, for example, micronutrients are recommended to be supplemented through protein substitutes; however, not all formulas are equally supplemented and some of them are not added with micronutrients. Data on pyridoxine and riboflavin status in these patients are particularly scarce. In long-chain fatty acid oxidation disorders, no specific recommendations on micronutrient supplementation are available. Regarding carbohydrate metabolism disorders, the difficult-to-ascertain sugar content in supplementation formulas is still a matter of concern. A ketogenic diet may predispose one to both oligoelement deficits and their overload, and therefore deserves specific formulations. In conclusion, our overview points out the lack of unanimous approaches to micronutrient deficiencies, the need for specific formulations for IMDs, and the necessity of high-quality studies, particularly for some under-investigated deficits.

## 1. Introduction

Trace elements and vitamins, called together “micronutrients”, are essential components of human nutrition in health and disease [1,2]. They play a variety of biochemical roles as cofactors and coenzymes in metabolism, as antioxidants, and in genetic regulation and protein folding, being crucial for maintaining tissue function and metabolism [3].

For the general population, international recommendations for micronutrient intake are available in the form of Recommended Dietary Allowances (RDA) or Dietary Reference Intakes (DRI), but the impact of micronutrient deficiencies in disease settings remains limited [4].

The roles of water-soluble vitamins in cellular metabolism have been clarified for many years. In fact, vitamin-derived cofactors intervene in a series of biochemical reactions and, consistently, their genetically determined deficiencies, at various levels, are linked to clinical pictures of variable severity, sometimes with serious and fatal results [5,6,7,8,9,10].

It should also be mentioned that inherited metabolic disorders (IMDs), when treated with a specific diet, may result in secondary vitamin deficiencies. The major therapy strategy for a significant number of IMDs essentially involves a specialized diet treatment, requiring the reduction or exclusion of certain macronutrients, focused on the enzyme deficiency causing the disorder [11,12].

On the one hand, this regimen makes it possible to reduce the effects of the enzymatic defect, significantly improving the clinical outcome of the disorder; on the other hand, a selective diet, especially at the growing age, could be associated with a reduced intake of micronutrients. To escape this problem, over the years, guidelines for the management of different IMDs have reported recommendations on the integration of vitamins and trace elements, aimed at reducing this nutritional risk [13,14,15,16].

Nevertheless, there are currently few data on the most common micronutrient abnormalities in different IMDs and there is still great heterogeneity in clinical practice regarding micronutrient supplementation.

## 2. Aim of the Study and Strategy Search

In this review, we aim to: (1) define which are the most commonly reported micronutrient deficits to which the different types of diet therapy predispose; (2) report the available evidence regarding the management of their deficiency; and (3) highlight the main critical points related to each nutritional supplementation.

A search was performed in PubMed/Medline and Embase to identify studies investigating the nutritional aspects of patients with IMDs undergoing dietotherapy. In particular, we focused on nutrient intake and nutritional deficiency related to vitamins and oligoelements. We searched for publications in English only. Every accessible publication published before 1 September 2023 was studied for this review.

## 3. Micronutrients, Their Nutritional Sources, and Their Function

The thirteen vitamins currently reported in human nutrition are divided into two categories, based on their relative solubility: water-soluble vitamins and fat-soluble vitamins [3]. 

The usefulness of the above classification mainly concerns the absorption and metabolic fate of vitamins taken with the diet [17,18]. Table 1 shows the most important vitamins and minerals for human health, along with their main characteristics, nutritional sources, biological biomarkers, and their deficiencies’ main clinical manifestations.

Many vitamins (especially those of group B) regulate hundreds of metabolic reactions by acting as coenzymes, contributing to energy-producing reactions and facilitating metabolic and physiological processes throughout the body [9,36,37,38,39].

The daily requirement of vitamins varies from species to species and from individual to individual, based on numerous factors (age, state of health, diet, sporting activity).

Due to their short stay in the body, a regular intake of water-soluble vitamins in the diet is necessary to avoid deficiencies. They are, for the vast majority, not accumulated in the body and are easily excreted in the urine. Humans have evolutionarily lost the ability to synthesize vitamins and have to obtain them from foods of animal and vegetal origin (except for vitamin B12) and to a lesser extent from the gut microbiota’s production [9,40,41].

Fat-soluble vitamins, thanks to their affinity for fats, are deposited in the liver and in the adipose tissue; as a result, the body can build up significant reserves of fat-soluble vitamins [42].

Trace elements are micronutrients that are needed in very small amounts through the diet but are critical for the prevention of acute and chronic diseases [43]. There are currently nine trace minerals for which humans are considered to have a nutritional requirement for, classified by the World Health Organization (WHO) [44]: iron, zinc, copper, selenium, iodine, manganese, molybdenum, chromium, and cobalt, with the first four being the most common mineral deficiencies.

Furthermore, because each essential trace element is linked to multiple enzymes, a deficiency of one of these elements can contribute to different metabolic abnormalities and clinical conditions. In particular, they are cofactors of a number of enzymes involved in the antioxidant system and in the body’s homeostatic mechanisms, especially inflammation and oxidative stress, which are vital for human health [45,46,47].

## 4. IMDs Requiring Special Diets

Nutritional therapy in IMDs is based on the basic principle of reducing the concentrations of toxic substrates by reducing the assumption of nutrients that produce them or by increasing their excretion while providing deficient products through supplementation. This approach is necessary for the normal growth and development of patients affected by several IMDs [48]. Special medical foods that include macro- and micronutrients but omit the offending substrate are available to help prevent such deficiencies. In addition to medical foods, other specialized nutritional products, including high doses of vitamins and amino acids, may be used in the management of IMDs. 

Table 2 reports the main IMDs requiring a special diet, distinguishing them by type of disorder and the principal category of limited food.

### 4.1. Disorders of Amino acid Metabolism

#### 4.1.1. Phenylketonuria (PKU)

Phenylketonuria (PKU) is a rare inherited metabolic disorder characterized by the partial or total inability to convert the essential amino acid Phenylalanine (Phe) into Tyrosine (Tyr) due to biallelic pathogenetic mutations of the liver enzyme phenylalanine hydroxylase (PAH). If PKU is detected at birth and treated with a Phe-restricted diet, the neurological sequaele secondary to Phe accumulation can be controlled [13]. 

**Figure 1:**
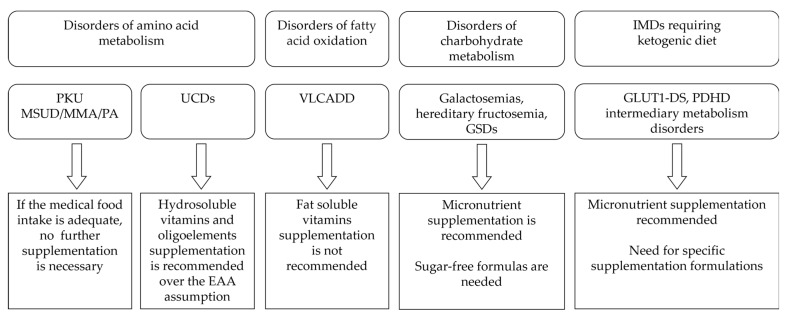
Main recommendations regarding micronutrient supplementation in different IMDs.

The Phe-restricted diet requires strict monitoring of patients’ nutritional status according to the PKU severity and type of diet [56,57]. The majority of patients, with the exception of those with mild hyperphenylalaninemia, consume little animal protein and mostly low natural protein diets. Therefore, supplemented Phe-free L-amino acids or formulations with no or little Phe content, such as Glycomacropeptides (GMP), are the main sources of micronutrients [58]. The necessary daily intake of micronutrients can be obtained by regularly assuming these formulations [13] (Figure 1). 

However, if the intake of Phe-free L-amino acid supplements is suboptimal, which is common in adolescence [58,59], the risk of micronutrient deficiency is higher, with iron, zinc, selenium, and vitamin B12 deficiency being particularly frequent in the PKU diet [13,60] (Table 3). 

However, clinical symptoms of micronutrient deficiency are rarely reported, being mainly described for vitamin B12 deficiency, particularly after reducing or stopping micronutrient supplements or Phe-free L-amino acid supplements while following a vegan-like diet [61,62]. 

Markers of micronutrient status in PKU patients, such as ferritin, hemoglobin, mean corpuscular volume (MCV) for iron, methylmalonic acid, and total serum homocysteine for vitamin B12, are useful to detect iron and vitamin B12 deficiency as their plasma concentrations are not fully related to their nutritional status [63,64] (Table 1).

Studies by Evans et al. [65] and de Almeida et al. [66] showed that more than 90% of treated patients had adequate and normal ferritin levels. Crujeras et al. reported lower-than-normal selenium levels in 95% of PKU patients [60].

A high prevalence of vitamin D deficiency has been reported in PKU patients by Kose et al. (53.57%) [67] and confirmed by other authors [68,69]. The same authors report adequate levels of vitamin A and zinc, with excess of folic acid, copper, and vitamin E (Table 3). Other studies have confirmed high folate levels in patients associated with the high folate content of Phe-free L-amino acid supplements [70,71]. The long-term consequences of folate overload in PKU patients have not been assessed. 

A study on the nutritional characteristics of adult PKU patients, according to their dietary adherence, reported that all patients in the adherent group met the Lower Reference Nutrient Intakes for the vast majority of micronutrients assessed. Nonadherent patients had significantly lower intakes of thiamine, riboflavin, niacin, vitamin B6, and phosphorus [72].

The literature review revealed poor data related to riboflavin and pyridoxine status in subjects undergoing a protein-restricted diet. Some old case series, by measuring the plasma pyridoxal 5′-phosphate (PLP), report on differences in pyridoxine metabolism in PKU children compared to healthy subjects, raising the need for personalized supplementation in this group of patients [73]. Children with PKU also showed an increase in the FAD effect and a concurrent decrease in glutathione reductase activity upon stopping group B vitamin therapy [74]. These findings are indicative of an inadequate riboflavin status. Since functional and direct biomarkers can be used in clinical practice to evaluate the levels of these two vitamins [75,76], it is necessary to reevaluate the patients’ pyridoxine and riboflavin status. 

#### 4.1.2. Maple Syrup Urine Disease, Propionic and Methylmalonic Acidemia

Maple syrup urine disease (MSUD), methylmalonic acidemia (MMA), and propionic acidemia (PA) are rare, autosomal recessive, multisystemic inborn errors of branched-chain amino acid metabolism, treated with a low-protein diet, precursor-free amino acid and/or isoleucine/valine supplementation [77]. 

The most recent guidelines for the above disorders emphasize the need for regular monitoring of micronutrient statuses to ensure adequate micronutrient intake [49]. As a matter of fact, most amino acid-free medical foods are supplemented with nutrients and micronutrients that may be deficient in a low-protein or low-precursor amino acid diet regimen. These formulas are usually supplemented with essential fatty acids, docosahexaenoic acid (DHA), vitamin D, vitamin A, calcium, iron, zinc, and selenium. Compliance with a full medical food prescription is important to meet these nutrient requirements [78]. 

Limited data exist for single or combined micronutrient deficiencies in actual clinical settings, with only one case series demonstrating intakes below the recommended levels for the great majority of vitamins and minerals [79]. In particular, the metabolic diet used in MSUD, MMA, and PA may be low in calcium and vitamin D levels, both of which are essential for bone health (Table 3).

Nutritional deficiencies have also been described for selenium and thiamine [80], secondary to the low animal protein intake. In addition, high-dose vitamin E and Coenzyme Q10 [49] are administered in order to prevent or treat optic neuropathy, which may alter visual acuity in MMA and PA patients [81,82]. 

In general, individuals who are compliant with medical foods supplemented with the recommended vitamins and minerals may not need additional supplementation (Figure 1). In contrast, those individuals who tolerate more intact protein and therefore need less medical food may need additional supplementation [83].

#### 4.1.3. Urea Cycle Disorders

Urea cycle disorders (UCDs) are a group of IMDs caused by a loss of function in one of the enzymes responsible for ureagenesis [84]. Long-term management of UCDs aims to prevent hyperammonemia and ensure normal development by the use of vitamin and mineral supplements, low-protein diets, essential amino acid supplements, and ammonia scavengers [15].

Supplementation is necessary for UCD patients on low-protein diets because of the risk of vitamin and mineral deficiencies, particularly iron, zinc, copper, calcium, and cobalamin [85,86]. 

In early-diagnosed patients, vitamin and mineral supplementations are generally started at weaning, in concomitance with milk intake reduction. Late-onset patients who are on a self-selected low-protein diet usually need vitamin and mineral supplements and regular dietary assessments [15]. 

Micronutrient plasma levels were investigated in very few studies, reporting conflicting data. The food intake evaluation has revealed an intake below the recommended values of at least one of the following micronutrients: calcium, magnesium, potassium, zinc, copper, manganese, iodine, and vitamin B12 (Table 3). In all patients, plasma essential amino acid (EAA) levels were, however, within normal limits [87].

In UCD patients, since EAA supplements do not contain enough micronutrients, these should be provided separately to prevent their deficiency [88] (Figure 1). 

### 4.2. Disorders of Fatty Acid Oxidation

Fatty acid oxidation disorders (FAOD) are a group of IMDs characterized by the defective transport or β-oxidation of fatty acids and are particularly involved in producing energy during fasting and stress episodes [89,90].

Patients affected by very-long-chain Acyl CoA dehydrogenase deficiency (VLCADD), one of the most severe forms of FAOD, undergo a dietary long-chain fatty acid restriction. Since they are susceptible to deficits in essential fatty acids and fat-soluble micronutrients [91], they should be evaluated for both. These patients may require supplementation with DHA or oils rich in essential fatty acids, such as linoleic acid and α-linoleic acid, to meet their nutritional needs. However, there are no reports regarding vitamin supplementation in subjects with long-chain fatty acid restriction. Although lower than normal levels of fat-soluble vitamins have been reported, recommendations for their supplementation cannot be made at this time [92].

### 4.3. Disorders of Carbohydrate Metabolism

#### 4.3.1. Galactosemias

Galactosemias are a group of four hereditary disorders of galactose metabolism [93]. The most common form is Galactosemia type 1 due to deficiency of Galactose 1-phosphate urydyltransferase (GALT), which catalyzes one of the four reactions in the Leloir pathway, which converts galactose into glucose [94]. Diet is the cornerstone of the treatment of galactosemias, aimed at minimizing galactose intake [95,96]. 

An annual dietary assessment of calcium and vitamin D intake with measurement of plasma total 25-OH-vitamin D levels is recommended. Both calcium and vitamin D should be supplemented as necessary, following the age-specific recommendations for the general population.

Supplementation with vitamin K might be beneficial when combined with an adequate intake of calcium and vitamin D, but currently there is not enough evidence to recommend the routine use of vitamin K [50].

#### 4.3.2. Hereditary Fructosemia

Dietary restriction of fructose, sucrose, sucralose, and sorbitol is the cornerstone of treatment for hereditary fructosemia (HF), an IMD caused by a deficiency in aldolase B (fructose-1,6-bisphosphate aldolase), which is responsible for the cleavage of fructose-1-phosphate [97]. Since fruit and vegetable intake is a dietary requirement, micronutrient deficiencies, particularly of water-soluble vitamins, are likely. However, there is great heterogeneity in vitamin supplementation practices among specialized centers.

In a recent report [51], most of the HF participants presented vitamin C (96.7%) and folate (90%) dietary intake below the recommended population reference. Up to 69% of the participants received vitamin C supplementation and 50% received folic acid supplementation. The amount of vitamin C supplementation correlated positively with correspondent plasma levels. Furthermore, non-supplemented HF patients were vitamin C deficient, with a statistically significant difference with respect to supplemented HF patients and healthy controls. Ensuring adequate vitamin supplementation in a disease requiring a reduction in fruit and vegetable intake is imperative [98]; supplementation with “sugar-free” multivitamin formulations is recommended.

#### 4.3.3. Glycogen Storage Disorders (GSDs)

Liver glycogenosis: GSDI and III, GSDVI, and liver GSDIXs are a group of rare conditions due to a genetic enzymatic defect in the metabolism of glycogen [99]. They have in common hepatomegaly and hypoglycemia and undergo an overlapping dietetic approach. Although there is no consensus regarding the restriction of sugars in the diet, sucrose (fructose and glucose) and lactose (galactose and glucose) are often limited or avoided [52]. The most common among GSDs is GSDI, in which, as a result of the deficiency of glucose-6-phosphatase, fructose and galactose are not metabolized to glucose-6-phosphate [100,101].

Restricting fruit, juice, and dairy foods impacts two entire food groups and renders the diet inadequate. Careful assessment and supplementation of micronutrients are therefore required to avoid nutrient deficiencies. In a recent study, 61.5% of patients with GSDI who were tested for 25-OH-vitamin D levels were found to have insufficient levels (<30 ng/mL), despite their reported good compliance with prescribed supplements [53]. 

The restricted nature of the diet, aimed at maintaining normoglycemia, may also result in poor intake of iron, vitamin B12, and folic acid. In liver GSDs and in particular in GSDI, a complete multivitamin with mineral supplementation is essential. Without appropriate supplements, these patients are at risk of a variety of nutritional deficiencies.

### 4.4. IMD Requiring Ketogenic Diet

A ketogenic diet (KD) is characterized by a diet with a low carbohydrate, high fat, and a defined or variable protein content [54]. There are two main types of KD: the classical diet, which uses long-chain triglycerides as its primary fat source, and the medium-chain triglyceride (MCT) diet, which allows more carbohydrate and protein because of the increased ketogenic potential of MCT [102].

KD represents the recommended treatment for pyruvate dehydrogenase complex (PDHc) deficiency and glucose transporter type 1 deficiency syndrome (GLUT1-DS) as it directly targets the underlying metabolic condition.

In other IMDs, mainly of intermediary metabolism, such as glycogen storage diseases and disorders of mitochondrial energy supply, supplementation with ketone bodies may ameliorate clinical symptoms and laboratory parameters [76,103,104]. 

Side effects have been classically reported, including specific micronutrient deficiencies in vitamin D and calcium, vitamin C, thiamine, and selenium [105,106,107]. The KD should be supplemented with vitamins, minerals, and trace elements, with plasma levels of micronutrients regularly measured [54]. At the moment, there are no specific supplements designed for the KD, and concerns have been raised about the most commonly used micronutrient supplement, containing high amounts of the fat-soluble vitamins A and E [55], which are naturally high in KDs as a result of its high fat content. 

A low intake of oligoelements such as zinc, selenium, and magnesium has also been reported. In a study on children on a classical KD, only 3 of the 28 micronutrients met the American dietary reference intakes [108], with zinc and magnesium particularly compromised [109]. However, Liu et al. [110] reported low levels of phosphorus and folate in otherwise normal micronutrient statuses. Close monitoring of micronutrient statuses in patients undergoing KD is therefore mandatory.

## 5. Discussion

An adequate vitamin and trace element homeostasis represents one of the cornerstones of the management of all IMDs, especially those undergoing diet therapy, as most of them can potentially expose patients to different forms of oligoelement abnormalities; therefore, close monitoring is always necessary.

However, the evidence of vitamin and mineral status, the type of supplementation to be adopted, and the clinical benefit of this supplementation is sometimes not univocal and derives mostly from case studies.

The beneficial effect of supplementation with high doses of vitamins in the treatment of IMD goes beyond the scope of this short review and is exhaustively reported elsewhere [111].

In amino acid metabolism disorders, particularly in PKU, due to the limited intake of natural protein, micronutrients are supplemented through protein substitutes to prevent overt nutritional deficiencies. However, despite apparent adequate supplementation, maintaining sufficient vitamin and mineral levels continues to be a challenge [48].

Many substitutes for PKU diet management contain vitamins and minerals according to guidelines for the required amount of micronutrients by age. Nevertheless, these recommendations do not take into account the reduced bioavailability or lack of nutrient interactions resulting from excluding entire food groups from the diet [48,112]. For this reason, serum levels of some micronutrients remain low despite adequate intake, indicating limited bioavailability [113]. 

Furthermore, patients who discontinue or reduce the intake of their protein substitute without a proportional increase in their natural protein intake are even more at risk for overt micronutrient deficiencies, particularly during the growing age [56]. 

Without micronutrient supplementation of medical foods, >70% of patients with PKU would have inadequate intakes of 11 micronutrients (biotin, choline, pantothenate, vitamins D and E, potassium, calcium, iodine, magnesium, selenium, and zinc). On the other hand, more than 90% of subjects would obtain adequate intake of vitamin A from natural foods alone due to high intakes of provitamin A carotenoids from green leafy vegetables, squashes, carrots, and tomatoes and do not require supplementation [114].

However, not all formulas are equally supplemented and some of them, due to the target age and type of diet, are not added with micronutrients. This, if combined with a strictly vegan-like diet, may increase the risk of deficiency and requires ad hoc supplementation. 

Nutrition management in FAOD is characterized by a low-fat and low-protein diet. This dietetic approach is potentially at risk of lowering their fat-soluble vitamin intake, having effects on immune regulation, vision, and bone health [115]. Nevertheless, no specific recommendations are so far available on micronutrient supplementation in this group of disorders and clinical practices are highly variable.

More of a consensus has been reached on the opportunity for supplementation in carbohydrate metabolism disorders. Galactosemia, hereditary fructosemia, and GSDI are treated by excluding entire groups of nutrients, thus necessitating regular micronutrient supplementation. Different approaches are nevertheless used in clinical practice [52,53,98], with formulations of which the sugar contents are sometimes unreliably reported or difficult to ascertain.

The ketogenic diet, a therapeutic approach for an increasing number of IMDs, has been shown to positively impact brain function and ketotherapeutics have been used in several conditions. Due to the restricted type of allowed nutrients, this approach may predispose one to both oligoelement deficits and their overload. 

A further potential benefit of adding supplementation of vitamins B12 and B6, and/or folic acid has been postulated due to their ability to reduce homocysteine, an independent risk factor of cognitive decline [116] which is common in the vast majority of IMDs. This dietetic approach would therefore benefit specific micronutrient formulations, which should derive from individual supplementation protocols.

As diet therapy is mandatory in the treatment of many IMDs, there have been concerns about nutritional deficiencies secondary to this therapeutic approach for many years, particularly during the growing age when they can predispose one to impairment of physical development, reduced cognitive function, and lower immunity [117]. For this reason, micronutrient supplementation for infants and children, mainly conveyed by amino acid formulas, is targeted and adapted to different ages by means of several special formulations available on the market [118]. 

The dietetic management of pregnant women affected by IMDs also deserves special mention. Pregnancy, once contraindicated for many IMDs, is in fact increasingly reported and represents a further challenge in the management of these disorders [119]. Metabolic adaptations to the demands of pregnancy determine higher requirements for micronutrients and changes in the metabolisms of macronutrients. A tailored management of this period, also through a targeted composition of the formulations used, may contribute to improving both maternal and fetus outcomes [120].

The use of medical foods, modified low-protein foods, amino acid supplements, and high doses of vitamins for individuals with IMDs is not an option but rather a medical necessity.

However, the strict requirements of different diet regimens deserve specific formulations that should be different from those used in the general population. In this context, a closer engagement of decision makers and stakeholders in health policy may represent an important methodology for improving clinical-based decisions to develop new technologies and identify future directions [121].

Overall, there is a suboptimal quality and level of evidence regarding the impact of nutritional supplements on the dietary management of IMDs.

More research is needed to understand the real prevalence of oligoelement abnormalities and the most effective supplementation approach in order to prevent the development of nutritional deficiencies. Moreover, other factors that contribute to vitamin and mineral abnormalities, like an altered microbiome, have gained attention in recent years [122,123,124]. Research showing specific alterations in IMDs receiving diet therapy should pave the way to the possibility of microbiome-based interventions in IMDs to improve micronutrient status. 

## 6. Conclusions

Despite improvements in the nutritional management of patients on diet therapy for different types of IMDs, our critical overview indicates the lack of unanimous approaches to micronutrient deficiencies, the need for specific formulations for IMDs, and the necessity of studies with high-quality evidence, particularly for some under-investigated deficits, with the final purpose of optimizing supplementation and harmonizing approaches.

## Figures and Tables

**Table 1:** Micronutrients’ main characteristics, monitoring, and deficiency manifestations.

Micronutrient	Main Function	RDA/DRI/AI	Dose of Supplementation Recommended	Nutritional Sources	Biomarkers	Deficiency Clinical Manifestation	Web Ref.
Vitamin A (retinoids and carotenoids)	Regeneration of visual pigment, maintenance of mucosal membranes, and immune function	300–900 mcg RAE/day	Rarely recommended	Some types of fish, such as herring and salmon. Beef liver and other organ meats. Green leafy vegetables and other green, orange, and yellow vegetables such as spinach, sweet potatoes, carrots, broccoli, and winter squash. Fruits, including cantaloupe, mangos, and apricots. Dairy products, such as milk and cheese. Fortified breakfast cereals, eggs	Serum retinol and retinyl esters in serum	Clinical manifestations are represented by the gradual development of night blindness, an increased frequency of infections, the development of xeroderma and follicular hyperkeratosis, xerophthalmia, and conjunctival xerosis	[19]
Vitamin B1 (thiamin)	Carbohydrate metabolism, ATP production	0.2–1.2 mg/day	3–5 mg/day up to 10 mg/day in severe deficiencies	Whole grains and fortified bread, cereal, pasta, and rice. Meat (especially pork) and fish. Legumes, seeds, and nuts	Red blood cells (RBC) or whole blood thiamine diphosphate (ThDP)	In its early stage, thiamine deficiency can cause weight loss, anorexia, confusion, short-term memory loss, muscle weakness, and cardiovascular symptoms. The most common effect is beriberi, characterized by peripheral neuropathy and wasting, which can lead to impaired sensory, motor, and reflex functions. Another common manifestation is Wernicke–Korsakoff syndrome, i.e., Wernicke’s encephalitis, characterized by peripheral neuropathy, and Korsakoff’s psychosis.	[20]
Vitamin B2 (riboflavin, vitamin G)	Essential component of 2 coenzymes, flavin mononucleotide (FMN) and flavin adenine dinucleotide (FAD), involved in energy production, cellular function, growth, and development, and metabolism of fats, drugs, and steroids	0.3–1.3 mg/day	5–10 mg/day up to 160 mg/day in severe deficiencies	Eggs, organ meats (such as kidneys and liver), lean meats, and low-fat milk. Some vegetables (such as mushrooms and spinach). Fortified cereals, bread, and grain products	Erythrocyte glutathione reductase activity test	Deficiency is manifested by oral buccal lesions (cheilosis, glossitis, and angular stomatitis) and seborrheic dermatitis of the face, trunk, and scrotum. Other manifestations are ocular itching, burning, dryness, corneal inflammation, and photophobia, and normochromic, normocytic anemia and marrow aplasia	[21]
Vitamin B3 (niacin, nicotinic acid, vitamin PP)	Converted into its main metabolically active form, the coenzyme NAD. It is involved in redox reactions	2–16 NEs/day	250–500 mg/day	Animal foods, such as poultry, beef, pork, and fish. Some types of nuts, legumes, and grains. Enriched and fortified foods, such as many breads and cereals	Urinary determination of the two major niacin metabolites, N-methyl-nicotinamide (NMN) and N-methyl-2-pyridone-carboxamide (2-Pyr), is used to determine niacin biomarker status	Diarrhea, dermatitis, and dementia, collectively known as “pellagra” or “the three D disease”, and even death (four D) if not recognized and treated promptly	[22]
Vitamin B5 (pantothenic acid)	Used for the synthesis of coenzyme A (CoA) and the citric acid cycle	1.7–5 mg/day	10 mg up to 1000 mg/day	Beef, poultry, seafood, and organ meats, eggs and milk, vegetables such as mushrooms (especially shiitakes), avocados, potatoes, and broccoli. Whole grains, such as whole wheat, brown rice, and oats, peanuts, sunflower seeds, and chickpeas	Whole blood and urine (24 h collection) are the sample matrices that have proven to be the most informative	Headache Fatigue Irritability, restlessness Disturbed sleep Nausea, vomiting, stomach cramps Numbness or burning sensation in hands or feet Muscle cramps	[23]
Vitamin B6 (pyridoxin)	In its active forms, pyridoxal 5′ phosphate (PLP) and pyridoxamine 5′ phosphate (PMP), it is involved in > 100 reactions, related to protein metabolism, carbohydrates, and lipids, biosynthesis of neurotransmitters and in maintaining normal levels of homocysteine; gluconeogenesis and glycogenolysis, immune function, hemoglobin	0.1–1.7 mg/day	6–50 mg/day up to 200 mg/day in severe deficiencies	Poultry, fish, and organ meats, potatoes and other starchy vegetables	Plasma levels of PLP correlate with pyridoxine intake and body stores and are recognized as a status biomarker	Microcytic anemia Skin conditions (seborrheic dermatitis with cheilosis and glossitis) Depression Confusion Lowered immunity, angular stomatitis	[24]
Vitamin B7 (biotin or vitamin H)	Coenzyme for five carboxylase enzymes, which are involved in the digestion of carbohydrates, synthesis of fatty acids, and gluconeogenesis	5–30 mcg/day	5–10 mg/day	Meat, fish, eggs, and organ meats (such as liver), seeds and nuts, certain vegetables (such as sweet potatoes, spinach, and broccoli)	Direct analysis of biotin in blood, serum/plasma, and urine (MS/MS) Indirect measurement: urinary excretion (24 h urine) of biotin and of metabolites produced by biotin-dependent carboxylases and related metabolic pathways (3-hydroxyisovaleric acid, 3-hydroxyisovalerylcarnitine). 3-hydroxyisovaleric acid and 3- Hydroxyisovalerylcarnitine biotinidase activity	Thinning hair Scaly skin rashes around eyes, nose, mouth Brittle nails	[25]
Vitamin B9 (folic acid)	Folate functions as a coenzyme or cosubstrate in single-carbon transfers in the synthesis of nucleic acids (DNA and RNA) and metabolism of amino acids. It plays the most important role in the conversion of homocysteine to methionine in the synthesis of S-adenosyl-methionine, in the methylation of deoxyuridylate to thymidylate, in the formation of DNA, and is required for proper cell division.	65–400 mcg DFE/day	1–5 mg/day	Beef liver, vegetables (especially asparagus, brussels sprouts, and dark green leafy vegetables such as spinach and mustard greens), fruits and fruit juices (especially oranges and orange juice), nuts, beans, and peas (such as peanuts, black-eyed peas, and kidney beans)	Levels of folate in serum/plasma or RBC	Most symptoms of folate deficiency overlap with those of cobalamin deficiency, i.e., megaloblastic anemia and pancytopenia, glossitis, angular stomatitis, oral ulcers, neuropsychiatric manifestations, including depression, irritability, insomnia, cognitive impairment, psychosis, anorexia, and fatigue	[26]
Vitamin B12 (cobalamin)	Vitamin B12 is required for the development, myelination, and function of the central nervous system, red blood cell formation, and DNA synthesis. It is a cofactor for two enzymes, methionine synthase and L-methylmalonyl-CoA mutase	0.4–2.4 mcg/day	100–1000 mcg IM/SC	Fish, meat, poultry, eggs, milk, and other dairy products. Clams and beef liver, enriched breakfast cereals, nutritional yeasts	Direct cobalamin levels measurement	Megaloblastic anemia—a condition of larger-than-normal-sized red blood cells and a smaller-than-normal amount; this occurs because there is not enough vitamin B12 in the diet or poor absorption Pernicious anemia—a type of megaloblastic anemia caused by a lack of intrinsic factor so that vitamin B12 is not absorbed Fatigue, weakness Nerve damage with numbness, tingling in the hands and legs Memory loss, confusion Dementia Depression Seizures	[27]
Vitamin C (ascorbic acid and ascorbates)	It is required for the biosynthesis of collagen, L-carnitine, and certain neurotransmitters. It is also involved in protein metabolism and it is an important physiological antioxidant. It plays an important role in immune function and improves the absorption of nonheme iron, the form of iron present in plant-based foods	15–90 mg/day	200 mg up to 2–3 g/day	Citrus fruits (such as oranges and grapefruit) and their juices, as well as red and green pepper and kiwifruit. Broccoli, strawberries, cantaloupe, baked potatoes, and tomatoes. Fortified foods and beverages	Assessment of vitamin C status can be determined from its concentration in either plasma or leukocytes	Scurvy, the hallmark disease of severe vitamin C deficiency, displays symptoms resulting from the loss of collagen that weakens connective tissues: skin spots caused by bleeding and bruising from broken blood vessels Swelling or bleeding of gums, and eventual loss of teeth Hair loss Delayed healing of skin wounds Fatigue, malaise Iron-deficiency anemia due to decreased absorption of non-heme iron	[28]
Vitamin D (cholecalciferol, ergocalciferol)	It is important for building and maintaining healthy bones and teeth. It reduces inflammation. It aids in the process of cell growth. It plays a role in immune function and supports muscle function and strength	10–20 mcg-day	400–1000 IU/day (infants) up to more than 10,000 IU/day (high-risk adults)	Butter and fatty cheeses, beef liver. Fortified milk, soy milk, oat milk, and almond milk. Fatty fish (like trout, salmon, tuna, and mackerel) and fish liver oils. Fortified food	Serum/plasma concentrations of total 25-hydroxyvitamin D (25-OHD), the sum of 25-OHD3 and 25-OHD2, are recognized as a valid biomarker for vitamin D status	Rickets Osteomalacia	[29]
Vitamin E (tocopherol and tocotrienols)	Antioxidants protect cells from the damaging effects of free radicals, which are molecules that contain an unshared electron. Free radicals damage cells and might contribute to the development of cardiovascular disease and cancer. Unshared electrons are highly energetic and react rapidly with oxygen to form reactive oxygen species	4–15 mg/day	15–25 mg/kg or mixed tocopherols 200 IU/day	Vegetable oils like wheat germ, sunflower, and safflower oils, corn and soybean oils. Nuts and seeds. Green vegetables, such as spinach and broccoli. Fortified food	Vitamin E status is determined by the quantification of tocopherol in blood plasma or serum	Retinopathy (damage to the retina of the eyes that can impair vision) Peripheral neuropathy (damage to the peripheral nerves, usually in the hands or feet, causing weakness or pain) Ataxia (loss of control of body movements) Decreased immune function	[30]
Vitamin K (vitamin K1 and K2)	It functions as a coenzyme required for the protein synthesis. It is involved in blood clotting, hemostasis, and bone metabolism	2–120 mcg/day	1–2 mg/day (infant) 90–120 mcg/day (adult)	Green leafy vegetables, such as spinach, kale, broccoli, and lettuce. Vegetable oils. Some fruits, such as blueberries and figs. Meat, cheese, eggs, and soybeans	The quantification of circulating phylloquinone (vitamin K1) in blood plasma or serum remains the most commonly used marker of vitamin K status, although it is mainly an indirect biomarker of short-term phylloquinone intake.	A longer time for blood to clot or a prolonged prothrombin time (as measured in a physician’s office) Bleeding Hemorrhaging Osteopenia or osteoporosis	[31]
Copper	It is required for adequate growth, cardiovascular integrity, lung elasticity, neovascularization, neuroendocrine function, and iron metabolism	200–900 mcg/day	4–8 mg/day	Beef liver and shellfish such as oysters. Nuts, seeds, and chocolate. Wheat-bran cereals and whole-grain products, potatoes, mushrooms, avocados, chickpeas, and tofu	Ceruloplasmin: 98% of circulating copper is bound to ceruloplasmin	Microcytic anemia, neutropenia, osteoporosis, and hair depigmentation (copper is essential for melanin synthesis)	[32]
Iron	It is involved in oxygen and lipid metabolism, in protein production, cellular respiration, and DNA synthesis	0.27–18 mg/day	150–200 mg/day	Lean meat, seafood, and poultry. Iron-fortified breakfast cereals and breads. White beans, lentils, spinach, kidney beans, and peas. Nuts and some dried fruits, such as raisins	Plasma iron, hemoglobin, mean red cell volume (MCV), transferrin, transferrin saturation, total iron binding capacity (TIBC), hepcidin, ferritin Bone marrow iron soluble transferrin receptor	Microcytic anemia and/or low ferritin levels	[33]
Selenium	It is an active immunomodulator and antioxidant. It takes part in thyroxine conversion to triiodethyronine in thyroid hormone biosynthesis. As a sperm antioxidant, it protects its motility and fertility. Selenium is a serious factor in the biological and antioxidant protection of vascular endothelium, of low-density lipoproteins, protection of DNA, chromosomes	15–55 mcg/day	60–100 mg/day	Seafood, meat, poultry, eggs, and dairy products. Breads, cereals, and other grain products	Whole blood or plasma/serum selenium concentration	Increased incidence and virulence of viral infections, cardiac and skeletal muscle myopathy, and skin and nail effects (selenium concentration <0.4 mmol/L (<32 mg/L))	[34]
Zinc	Structural, catalytic, and intracellular and intercellular signaling component	2–11 mg/die	20–40 mg/day	Oysters, meat, fish, poultry, seafood such as crab and lobsters, and fortified breakfast cereals. Beans, nuts, whole grains, eggs, and dairy products	Whole blood, plasma, serum, urine	Alopecia, skin rash of face, groin, hands, and feet, growth retardation, delayed sexual development and bone maturation, impaired wound healing and immune function, diarrhea, and blunting of taste and smell	[35]

RDA/DRI/AI (Recommended dietary allowances/Dietary Reference Intakes/Adequate Intakes); DFE (dietary folate equivalent) is defined as 1 mg DFE = 1 mg food folate = 0.6 mg folic acid from fortified food or a supplement consumed with food = 0.5 mg of a folic acid supplement taken on an empty stomach or provided via IV. RAE (retinol activity equivalents) 1 mcg/RAE = 1 mcg retinol, 2 mcg supplemental beta-carotene, 12 mcg dietary beta-carotene, or 24 mcg dietary alpha-carotene or beta-cryptoxanthin. NE (niacin equivalents) = 1 milligram of niacin or 60 mg of tryptophan. IM (intramuscular) SC (subcutaneous). IU VIT D (international unit of vit D) = 1 IU of vitamin D is equivalent to 0.025 micrograms.

**Table 2:** IMDs requiring special diet and associated micronutrient deficiency.

Category of Disorder	Type of Disorder	Diet Regimen	Principal Category of Limited Food	Ref.
Amino acid disorders	Phenylketonuria	Low Phenylalanine intake	Meat, fish, eggs, pulses, milk and dairy products, cereals	[13]
	Organic acidosis (OA)	Low natural protein intake	Meat, fish, eggs, pulses, milk and dairy products, cereals	[14,49]
	UCDs	Low natural protein intake	Meat, fish, eggs, pulses, milk and dairy products, cereals	[15]
Fatty acid oxidation disorders	VLCADD	Low intake of long-chain fatty acids	Full-fat and semi-skimmed milk, egg yolks, fatty fish and meat, cheese, butter, margarine, vegetable oil, dried fruit, oilseeds, chocolate, baked products, industrial products	[16]
Carbohydrate disorders	Galactosemia	Galactose-restricted diet	Milk and derivatives	[50]
	Hereditary fructose intolerance	Minimal fructose and absolute exclusion of sucrose and alimentary additives like caramel (E150), sweeteners isomalt (E963), maltitol (E965) mannitol (E421), sorbitol (E420), xylitol (E967) intake	Fruit, honey, vegetables, other products containing sugar	[51]
	Glycogen storage disorders (I, III, VI, IX)	Fructose, sucrose, and galactose exclusion (I) Moderately high protein and low sugar intake (III) Low carbohydrate intake (VII)	Fruit, honey, vegetables, products containing sugar	[52,53]
IMD treated with ketogenic diet	GLUT1 deficiency PDH deficiency	Low carbohydrates and high fat intake	Fruit, dessert pastry, sweets, juice, pasta, cereals and baked products, potatoes, pulses	[54,55]

**Table 3:** Panoptic vision of the reported micronutrient abnormalities in IMDs.

		Category of Disorders							
		Amino acid disorders			Fatty acid oxidation disorders	Carbohydrate disorders			IMDs treated with KD
		Type of disorder							
		PKU	OAs	UCDs	VLCADD	Galactosemias	HF intolerance	GSDs (I, III, VI, IX)	GLUT1-D PDH-D
**Vitamins**	A								
	B1								
	B2								
	B3								
	B5								
	B6								
	B7								
	B9								
	B12								
	C								
	D								
	E								
	K								
**Minerals**	Ca								
	Cu								
	K								
	Fe								
	I								
	Mg								
	Mn								
	Se								
	Zn								

							High micronutrient level		

							Low micronutrient level		

## Data Availability

The data presented in Table 1 are openly available on [https://ods.od.nih.gov/factsheets/list-all/, accessed on 28 November 2023].

## Abbreviations

DRI	dietary reference intakes
EAA	essential amino acid
FAD	flavin adenine dinucleotide
FAOD	fatty acid oxidation disorder
FMN	flavin mononucleotide
GALT	galactose 1-phosphate urydyltransferase
GLUT1-DS	glucose transporter type 1 deficiency syndrome
GMP	glycomacropeptides
GSD	glycogen storage disorder
HF	hereditary fructosemia
IMD	inherited metabolic disorder
KD	ketogenic diet
LCFAOD	long-chain fatty acid oxidation disorder
MCT	medium-chain triglyceride
MCV	mean corpuscular volume
MMA	methylmalonic acidemia
MSUD	maple syrup urine disease
NE	niacin equivalents
OA	organic acidosis
PA	propionic acidemia
DHA	docosahexaenoic acid
PAH	phenylalanine hydroxylase
PDHc	pyruvate dehydrogenase complex
PKU	phenylketonuria
PLP	pyridoxal 5′-phosphate
RDA	recommended dietary allowances
UCD	urea cycle disorder
VLCADD	very-long-chain Acyl CoA dehydrogenase deficiency
WHO	World Health Organization
